# A Comprehensive Review of Motherhood and Mental Health: Postpartum Mood Disorders in Focus

**DOI:** 10.7759/cureus.46209

**Published:** 2023-09-29

**Authors:** Anushree Modak, Vaishnavi Ronghe, Kavita P Gomase, Manjusha G Mahakalkar, Vaishali Taksande

**Affiliations:** 1 Obstetrics and Gynaecology, Srimati Radhikabai Meghe Memorial College of Nursing, Datta Meghe Institute of Higher Education & Research, Wardha, IND

**Keywords:** preventive measures, cultural influences, stigma reduction, intervention strategies, maternal mental health, postpartum mood disorders

## Abstract

The journey of motherhood encompasses a profound array of emotions, experiences, and challenges that extend beyond the surface of joy and elation. This review delves into the crucial yet often underexplored realm of postpartum mood disorders, aiming to illuminate their significance and foster understanding. Postpartum mood disorders, including postpartum depression, anxiety disorders, and psychosis, impact the mental well-being of mothers during a pivotal phase of their lives. Through a comprehensive exploration, this review elucidates the various dimensions of these disorders, from their definitions and classifications to their prevalence and impact on both mothers and families. Identifying and diagnosing postpartum mood disorders is discussed in detail, shedding light on the emotional, cognitive, and physical symptoms that warrant attention. Screening and assessment tools are highlighted as essential instruments for early detection, while challenges in diagnosis, including the overlap with typical postpartum experiences and the influence of stigma, are explored. The review further delves into treatment and intervention, underscoring the importance of psychotherapy, pharmacological interventions, and individualised treatment plans. The roles of healthcare providers and mental health professionals in offering support and guidance are emphasised, emphasising the significance of a collaborative approach. Cultural and societal influences are crucial in shaping perceptions of motherhood and mental health. The review explores how these influences can create barriers to seeking help and highlights the importance of destigmatising postpartum mood disorders. It underscores the urgency of raising awareness and fostering a supportive environment that empowers mothers to seek assistance without fear of judgment. Looking toward the future, the review points to potential research directions, such as advances in understanding hormonal influences and exploring the long-term effects on maternal mental health. The overarching call to action resonates - increased awareness, support, and dismantling stigma are imperative. A hopeful vision is presented: a future where all mothers receive appropriate mental health care, no mother stands alone in her motherhood journey, and societal understanding and compassion thrive.

## Introduction and background

Motherhood is a transformative journey that brings joy, challenges, and profound changes to a woman’s life. Amid the joyous moments and new beginnings, it is essential to acknowledge the less-discussed aspects of motherhood, particularly those related to mental health. This review delves into a critical dimension of motherhood-postpartum mood disorders and sheds light on their significance, highlighting the pressing need to address mental health during this pivotal phase of a woman’s life [1].

The transition to motherhood is marked by hormonal fluctuations, sleep deprivation, and adjustments to a new role, potentially impacting a woman’s mental well-being. Postpartum mood disorders, including postpartum depression (PPD), anxiety disorders, and even rare but severe cases of postpartum psychosis, can cast a shadow over what is meant to be a joyous time. The significance of this topic lies in the potential long-term consequences of untreated postpartum mood disorders, affecting not only the mother but also her family unit [2].

Maternal mental health is not just an individual concern but has far-reaching implications for the child’s emotional, cognitive, and social development. Research has established the interplay between a mother’s mental well-being and her ability to provide responsive caregiving. Unaddressed postpartum mood disorders can disrupt the formation of a secure mother-child attachment and hinder the child’s emotional regulation and overall mental health. Furthermore, the effects of maternal mental health reverberate through the family, influencing partner relationships, sibling dynamics, and the overall family environment [3].

The purpose of this review is twofold: first, to deepen our understanding of postpartum mood disorders by exploring their various forms, causes, and prevalence, and second, to emphasise the significance of timely intervention and support for mothers experiencing these disorders. By delving into the complexities of these disorders, we aim to provide healthcare practitioners, mental health professionals, families, and society with insights into practical strategies for prevention, early detection, and management. Additionally, we will explore the role of cultural and societal factors in shaping perceptions of maternal mental health and help bridge the gap between awareness and destigmatisation.

## Review

Understanding postpartum mood disorders

Definition and Classification of Postpartum Mood Disorders

The spectrum of postpartum mood disorders encompasses a range of emotional and psychological challenges that can affect mothers after childbirth. These disorders are not only diverse in their manifestations but also differ in their severity and duration [2]. The following classifications shed light on the multifaceted nature of these disorders:

Postpartum depression (PPD): PPD is characterised by persistent sadness, hopelessness, and a loss of interest or pleasure in previously enjoyed activities. It often manifests within the first few weeks after childbirth but can emerge up to a year later. Physical symptoms such as changes in appetite and sleep patterns might accompany emotional distress [2].

Postpartum anxiety disorders: These disorders encompass a range of anxiety-related conditions, including generalised anxiety disorder (GAD), panic disorder, and obsessive-compulsive disorder (OCD). New mothers with postpartum anxiety may experience excessive worry, restlessness, and intrusive thoughts about their baby’s safety [4].

Postpartum psychosis: Although relatively rare, postpartum psychosis is a severe disorder characterised by hallucinations, delusions, and disorganised thinking. It typically emerges within the first few weeks after childbirth and requires immediate medical intervention [5].

Baby blues vs. more severe disorders: Distinguishing between the “baby blues” and more severe postpartum mood disorders is crucial. Baby blues are common, short-lived mood fluctuations affecting up to 80% of new mothers and generally resolve independently within a couple of weeks. In contrast, postpartum mood disorders involve persistent and often worsening symptoms that require professional attention [2].

Prevalence and Statistics

Postpartum mood disorders are more prevalent than commonly perceived. PPD alone affects around 10-20% of new mothers worldwide. Postpartum anxiety disorders and psychosis are less common but still demand attention due to their potential severity. The prevalence varies across cultures, highlighting the complex interplay of biological, psychological, and sociocultural factors [6].

Risk Factors and Contributing Factors

Hormonal changes: The journey of pregnancy and childbirth is accompanied by dramatic hormonal shifts. The abrupt decline in oestrogen and progesterone levels after delivery is of particular significance. These hormonal changes can profoundly impact mood regulation mechanisms within the brain. The intricate interrelationship between these hormonal fluctuations and neurotransmitter activity can potentially render individuals susceptible to mood disorders during the postpartum period. The abruptness of these hormonal changes further underscores their potential influence on mood regulation [7].

Psychological factors: Personal mental health history plays a pivotal role in developing postpartum mood disorders. Individuals with a prior history of depression or anxiety may exhibit heightened vulnerability during this phase. Pregnancy and childbirth’s physiological and psychological challenges can act as triggers, exacerbating these preexisting tendencies. Furthermore, unresolved emotional issues, compounded by the stress and uncertainty of new motherhood, can fuel the onset of mood disorders. Unrealistic expectations surrounding the experience of motherhood, often shaped by societal norms and personal perceptions, can add another layer of psychological strain [8].

Social support and environment: The support network and environment in which a new mother finds herself are influential determinants of her mental well-being. Adequate social support can act as a protective buffer against developing postpartum mood disorders. Conversely, a lack of support or strained relationships can magnify a new mother’s challenges, making her more vulnerable to emotional distress. Isolation, whether physical or emotional, can compound the risk, as it restricts the availability of sources to seek comfort, understanding, and guidance. The absence of a strong support network can amplify the feelings of inadequacy and overwhelm that often accompany the transition into motherhood [9].

Impact on mothers and families

Effects on Maternal Well-being

Emotional distress and mood fluctuations: Mothers grappling with postpartum mood disorders often find themselves caught in a tumultuous whirlwind of emotions. These emotions can span from overwhelming sadness that engulfs their days to heightened irritability and restlessness. The unpredictable mood fluctuations can be likened to an emotional rollercoaster, rendering even simple tasks a challenge and robbing them of the ability to savour life’s ordinary moments. The weight of these emotions can be paralysing, making it difficult to engage in daily activities, interact with loved ones, and find joy in things that once brought happiness [2].

Impact on self-esteem and self-image: Postpartum mood disorders can profoundly distort a mother’s sense of self. The internal dialogue becomes clouded by negative self-perceptions, leading to a sense of inadequacy and self-doubt. Mothers may internalise their struggles as personal failings, viewing themselves as incapable of meeting motherhood’s demands. This negative self-image can magnify emotional challenges, forming a self-reinforcing cycle that perpetuates emotional distress [10].

Relationship with the baby and partner: One of the most poignant consequences of postpartum mood disorders is their impact on the mother’s relationship with her infant and her partner. Emotional distress can hinder a mother’s ability to form a strong, nurturing bond with her baby, impairing her capacity to provide the responsive care crucial for the infant’s healthy development. This can lead to feelings of guilt and inadequacy, further intensifying her emotional turmoil. Simultaneously, strained emotional states can reverberate into the mother’s partnership, causing communication breakdowns and emotional distance. Partners might struggle to comprehend the changes they observe, leading to a sense of helplessness in providing support [11].

Effects on Family Dynamics

Role of the partner and extended family: Partners emerge as critical sources of support during the postpartum period, offering understanding, assistance, and emotional connection. However, the challenges posed by a mother’s mood disorder can create strains that ripple across the family unit. The partner’s emotional well-being might be impacted as they navigate how to best provide the necessary care and support while managing their emotional responses. Equally important are extended family members, whose empathy and involvement can significantly lighten the load on the mother. Their understanding and contributions can help create a more conducive environment for the mother’s recovery and family well-being [12].

Sibling relationships and family bonding: Families with older children face unique challenges when a mother struggles with a postpartum mood disorder. These struggles can impact her capacity to engage fully with all her children, leading to feelings of neglect or even resentment among her siblings. The emotional turbulence can affect the overall family bonding experience, potentially introducing tensions and imbalances that disrupt the equilibrium of familial relationships [13].

Long-term implications if left untreated: The consequences of untreated postpartum mood disorders can cast long shadows, profoundly affecting not only the mother but also the development and dynamics of her family. Children raised by mothers grappling with unaddressed mood disorders might encounter developmental delays from disrupted caregiving and emotional engagement. These children could also face difficulties regulating their emotions and may have a heightened vulnerability to their mental health issues later in life. The foundations of family dynamics might be reshaped as the mother’s unmet emotional needs continue reverberating, potentially leading to persistent imbalances and strained relationships in the long run [11].

Identifying and diagnosing postpartum mood disorders

Recognising Symptoms

Accurate identification of postpartum mood disorders is paramount as it is the foundation for timely and effective intervention. By recognising the myriad symptoms that encompass these disorders, healthcare professionals can facilitate early diagnosis and thereby prevent potential escalation of the condition.

Emotional symptoms: The emotional toll of postpartum mood disorders can be profound and wide-ranging. Mothers grappling with these disorders might find themselves immersed in persistent sadness, emptiness, or hopelessness that extends beyond what is commonly expected during the postpartum period. Unexplained and uncontrollable crying spells and intense irritability are further indications of emotional distress. A particularly concerning manifestation is emotional numbness, where mothers might describe feeling disconnected from themselves, their surroundings, or even their newborns [2].

Cognitive symptoms: Postpartum mood disorders often cast a shadow on cognitive functioning. Mothers might experience notable difficulty in concentrating on tasks, making decisions, or even thinking clearly. This cognitive fog can exacerbate feelings of frustration and inadequacy. A striking hallmark is the emergence of intrusive thoughts - distressing and unwanted ideas or mental images that might be distressing or terrifying. These thoughts might revolve around themes of harm to the baby or oneself despite having no intent or desire for such actions [14].

Physical symptoms: The physical toll postpartum mood disorders take on mothers is tangible and impactful. Overwhelming fatigue, beyond the expected sleep deprivation associated with caring for an infant, is a common symptom. Changes in appetite and sleep patterns can further compound the physical strain. Many mothers may also experience unexplained physical aches and pains, which often intensify their already grappling with emotional distress. Notably, losing interest in previously enjoyable activities can be a critical indicator that something needs to be corrected [15].

Screening and assessment tools

Screening and assessment tools constitute a fundamental component in the landscape of postpartum mood disorders. In the delicate period following childbirth, when the emotional well-being of mothers is especially vulnerable, these tools act as essential gateways to early detection and intervention. Two prominent examples of such tools, the Edinburgh Postnatal Depression Scale (EPDS) and the Postpartum Depression Screening Scale (PDSS), are crucial instruments in this endeavour [16].

The EPDS is widely recognised as a reliable self-report questionnaire that aids healthcare providers in identifying mothers who might be at risk of PPD. The EPDS is designed to explore various mood dimensions, including emotional symptoms such as sadness, anxiety, and irritability. By evaluating responses to specific questions, the EPDS quantifies the severity of these symptoms, effectively gauging the likelihood of a mother experiencing depressive symptoms. Its user-friendly format allows for straightforward administration and interpretation, making it a valuable tool for healthcare providers [17].

The PDSS is another notable instrument tailored to assess PPD. The PDSS encompasses a broader range of emotional, cognitive, and physical symptoms. Through carefully crafted questions, it captures the nuances of a mother’s emotional experience, aiding healthcare providers in understanding the complexity of her emotional state. This multidimensional approach enhances assessment accuracy, ensuring a more comprehensive evaluation of a mother’s mental well-being [18].

Both of these screening tools serve a dual purpose. They identify mothers who might be at risk of postpartum mood disorders, thereby facilitating timely intervention. Furthermore, they offer a crucial means of differentiation-helping healthcare providers distinguish between the more transient “baby blues” and the potentially more severe mood disorders that demand specialised attention.

Challenges in Diagnosis

Overlap with typical postpartum experiences: One of the primary challenges lies in the overlap between symptoms of postpartum mood disorders and those considered within the realm of typical postpartum experiences. Fatigue, mood swings, and appetite changes are expected postpartum. Distinguishing between transient mood changes often accompanying the adjustment to new motherhood and persistent symptoms indicative of a disorder requires a nuanced understanding. Healthcare providers must carefully evaluate these symptoms’ duration, intensity, and impact to make an accurate diagnosis. A clear differentiation is vital to avoid either underdiagnosis, which may lead to untreated disorders, or overdiagnosis, which could cause unnecessary distress [19].

Stigma and cultural considerations: Stigma surrounding mental health issues can create substantial barriers to accurate diagnosis. Women experiencing postpartum mood disorders might be hesitant to acknowledge their struggles due to the fear of judgment or societal misconceptions about mental health. This reluctance to seek help delays intervention and exacerbates the emotional burden. Additionally, cultural norms and expectations related to motherhood vary widely across societies. Some cultures idealise the image of the “strong” and “selfless” mother, which might discourage women from admitting their emotional challenges. Cultural differences can also influence how symptoms are perceived and reported. Expressions of distress might be framed differently or downplayed due to cultural norms, leading to potential inaccuracies in diagnosis [19].

Treatment and intervention

Psychotherapy Options

Psychotherapy emerges as a fundamental cornerstone in the comprehensive treatment of postpartum mood disorders, pivotal in addressing the intricate emotional landscape experienced by new mothers. This therapeutic approach offers a range of practical strategies designed to alleviate emotional distress and foster overall well-being, tailored to the unique needs of each individual.

Cognitive-behavioural therapy (CBT): Among the prominent psychotherapeutic modalities, CBT stands out as a structured and goal-oriented approach. CBT operates on the premise that our thoughts, emotions, and behaviours are interconnected. CBT provides a roadmap for identifying negative thought patterns and behaviours contributing to emotional distress for mothers grappling with postpartum mood disorders. Mothers learn to challenge and reframe these negative cognitions through collaborative work with trained therapists, replacing them with healthier and more constructive alternatives. This empowerment equips them with valuable coping skills, enabling them to manage their symptoms more effectively and cultivate a more optimistic outlook on their journey through motherhood [20].

Interpersonal therapy (IPT): IPT is another instrumental psychotherapeutic approach tailored to the challenges of postpartum life. It recognises the profound shifts in interpersonal relationships accompanying motherhood and targets these dynamics as a central focus of therapy. IPT assists mothers in enhancing their communication skills and improving their interpersonal relationships. It equips them with tools to navigate the changes that motherhood introduces into their relationships with partners, family members, and friends. By addressing these shifts head-on, IPT empowers mothers to strengthen their support systems and cultivate healthier, more fulfilling relationships, thus alleviating some of the emotional burdens associated with postpartum mood disorders [21].

Support groups and peer counselling: In addition to individual therapy modalities, the power of communal support cannot be underestimated. Support groups and peer counselling offer a unique and invaluable space for mothers to connect, share their experiences, and find solace in knowing they are not alone in their struggles. Support groups provide a haven for mothers to express their thoughts and feelings without judgment, receiving validation and understanding from peers who have traversed similar challenges. This shared journey helps reduce feelings of isolation and provides a platform for learning effective coping strategies from those who have firsthand experience. Peer counselling, within this context, fosters an even more profound sense of connection and empathy as mothers provide support for one another guided by their shared experiences [22].

Pharmacological Interventions

Pharmacological treatments play a crucial role in managing postpartum mood disorders, particularly in cases where symptoms are severe or unresponsive to psychotherapy. Among the most commonly prescribed pharmacological interventions are antidepressants and anti-anxiety medications, with Selective Serotonin Reuptake Inhibitors (SSRIs) taking the forefront. These medications regulate neurotransmitter levels in the brain, effectively mitigating emotional distress and ameliorating the symptoms associated with PPD and anxiety [15].

However, initiating pharmacological treatment is not without careful consideration, particularly for breastfeeding mothers. Healthcare providers face the delicate task of balancing the potential benefits of medication with the potential risks posed to both the mother and the infant through breast milk. This complex decision-making process considers several factors, including the severity of the mother’s condition, the specific medication’s safety profile for breastfeeding, and the potential impact on the baby’s development [23].

It’s important to note that many antidepressants and anti-anxiety medications are deemed safe for breastfeeding mothers. This assurance stems from extensive research that has evaluated the passage of these medications into breast milk and their potential effects on infants. In this context, healthcare providers are pivotal in guiding mothers through this decision-making process, providing information about the benefits and potential risks, and working collaboratively to determine the most suitable course of action for the mother’s well-being and the baby’s health.

Importance of Individualized Treatment Plans

Recognising the unique nature of each mother’s experience when dealing with postpartum mood disorders is paramount in providing adequate care. The cookie-cutter approach doesn’t suffice, given the broad spectrum of symptoms, backgrounds, and circumstances that mothers bring to the table. This is where the significance of individualised treatment plans comes into play [24].

Understanding the complexity: Postpartum mood disorders can manifest differently in each woman. Some might primarily experience emotional turmoil, while others struggle with cognitive symptoms or physical manifestations. Furthermore, factors such as the severity of symptoms, personal medical history, and existing support networks all contribute to the complexity of each case [14].

Personalised assessment: An effective treatment plan starts with a thorough and personalised assessment. Healthcare professionals must delve into the specific symptoms a mother faces, her medical history (including any preexisting mental health conditions), her personal preferences, and the context in which she’s navigating motherhood [25].

Tailored approaches: Psychotherapy and pharmacological interventions are powerful tools, but their efficacy dramatically depends on how well they align with an individual’s needs. Some mothers might resonate more with CBT, while others might find relief through IPT. Similarly, when it comes to medications, considering factors like potential side effects, breastfeeding compatibility, and personal comfort with medication use is crucial [26].

Maximising outcomes: An individualised approach considers each mother’s specific symptoms and needs and ensures that treatment is more likely to be accepted and adhered to. When mothers feel their care plan respects their uniqueness and aligns with their values, they are more likely to engage wholeheartedly in the healing process [26].

Holistic well-being: Motherhood is a multidimensional journey beyond just the emotional realm. It encompasses physical changes, shifts in roles and responsibilities, and adjustments to daily routines. An individualised treatment plan acknowledges and addresses these interconnected aspects, aiming to restore holistic well-being [27].

The power of collaboration: The beauty of individualised treatment plans lies in the collaboration between healthcare professionals and mothers. By engaging mothers in the decision-making process and valuing their input, the treatment plan becomes a joint effort to achieve better mental health outcomes [28].

Role of Healthcare Providers and Mental Health Professionals

The role of healthcare providers and mental health professionals is of paramount importance in addressing postpartum mood disorders. This multidisciplinary approach brings together the expertise of various professionals to ensure the well-being of both mothers and their newborns [29].

Healthcare providers, encompassing obstetricians, paediatricians, and general practitioners, are often the frontline observers during the postpartum period. Routine postpartum check-ups provide crucial opportunities to assess a mother’s mental well-being alongside her physical health. These encounters allow healthcare providers to establish open lines of communication, creating an environment where mothers feel comfortable sharing their emotional experiences. By incorporating mental health assessments into routine postpartum care, healthcare providers can effectively identify early signs of distress or disorder, enabling timely intervention [30].

Mental health specialists, mainly those skilled in perinatal and postpartum care, offer a unique and specialised support layer. Their expertise is finely attuned to the emotional intricacies of the transition to motherhood. With comprehensive knowledge of the hormonal, psychological, and sociocultural factors, these specialists possess the insight to diagnose and treat postpartum mood disorders accurately. Their guidance is instrumental in tailoring treatment plans, whether that involves psychotherapy, pharmacological intervention, or a combination of both [31].

Moreover, mental health professionals provide a safe space for mothers to discuss their feelings, fears, and challenges openly. The non-judgmental and empathetic environment they foster encourages mothers to seek help without stigma. This therapeutic alliance between mental health professionals and mothers ensures that emotional struggles are met with understanding and that appropriate strategies for managing and overcoming these challenges are implemented [32].

Preventive measures

Prenatal Education and Preparation

Engaging in proactive measures during pregnancy can be pivotal in mitigating the risk of postpartum mood disorders. Among these measures, prenatal education is a cornerstone, offering expectant mothers a valuable tool to enhance their emotional resilience and well-being.

Prenatal education is a compass, guiding mothers through the labyrinth of emotions that can arise postpartum. This educational process equips expectant mothers with a comprehensive understanding of the emotional challenges that might await them after childbirth. It’s an opportunity to shed light on the diverse feelings they might encounter, ranging from the elation of new motherhood to the potential strains and anxieties that can emerge. With this knowledge, mothers are better prepared to recognise the natural variations in their emotional states, helping them avoid unnecessary distress or confusion [33].

Crucially, prenatal education empowers mothers by equipping them with strategies to manage and navigate these emotions effectively. Understanding that it’s entirely normal to experience a range of positive and challenging feelings reduces the stigma often associated with mood fluctuations. Expectant mothers can learn practical coping techniques like relaxation exercises, mindfulness practices, and stress-reduction strategies. This arsenal of coping tools enhances their emotional well-being and equips them to respond constructively to the emotional challenges that can emerge during the postpartum period [34]. Furthermore, prenatal education offers a roadmap to creating a solid and supportive postpartum network. Mothers gain insights into the importance of seeking assistance, not as a sign of weakness, but as a proactive step towards ensuring their well-being. They become familiar with the available resources, from mental health professionals to support groups, enabling them to reach out for help without hesitation when needed. By understanding where to turn and who to talk to, mothers are better positioned to seek timely support, preventing isolated struggles from escalating into more severe mood disorders.

Lifestyle Modifications

Exercise and physical well-being: Engaging in regular physical activity during both pregnancy and postpartum holds the potential for significant positive impacts on mental well-being. Exercise acts as a natural mood enhancer, promoting the release of endorphins, commonly referred to as “feel-good” hormones. These endorphins help alleviate feelings of stress, anxiety, and even depression. Engaging in activities such as yoga, walking, swimming, or other low-impact exercises helps maintain physical fitness and contributes to mental rejuvenation [35].

Moreover, exercise offers more than just a physiological boost. It can provide a valuable opportunity for new mothers to engage in self-care and carve out time for themselves amidst their caregiving responsibilities. Participating in group classes or outdoor activities can also foster social interaction, combating feelings of isolation that some mothers might experience during this transition period. By incorporating regular physical activity into their routines, new mothers can cultivate a sense of empowerment, confidence, and emotional resilience [36].

Nutrition and sleep: The significance of maintaining a balanced diet during the postpartum period cannot be overstated. Adequate nutrition provides the necessary fuel for physical recovery, especially after the demands of childbirth. But beyond physical health, a balanced diet rich in nutrients can directly impact emotional well-being. Certain nutrients, such as omega-3 fatty acids and B vitamins, are linked to improved mood regulation and mental clarity [37].

Equally crucial is prioritising sufficient sleep. New mothers face sleep deprivation due to the demands of round-the-clock care for their newborns. However, deliberate efforts to improve sleep quality can substantially affect mental health. Even if it means catching naps during the day, establishing a consistent sleep schedule can help regulate circadian rhythms and promote better sleep patterns. Creating a conducive sleep environment by minimising noise, dimming lights, and ensuring comfort further supports better sleep quality [38].

Social Support Systems

Partner involvement: The involvement of partners in a mother’s journey through postpartum mood disorders is paramount. Partners not only provide a vital emotional anchor but also offer practical assistance that can significantly alleviate the challenges faced by new mothers. Open and honest communication between partners fosters an environment where mothers feel comfortable expressing their feelings and seeking help when needed. Sharing responsibilities in caregiving, household chores, and other daily tasks can significantly reduce the burden on the mother, allowing her the time and space to focus on her well-being and recovery. Partners who consciously try to understand the emotional turmoil accompanying postpartum mood disorders strengthen the relationship, forging a more profound bond built on empathy and mutual support [9].

Family and friends as a support network: The presence of a robust support network, comprising not only partners but also extended family and friends, can serve as a lifeline for mothers grappling with postpartum mood disorders. People willing to lend a listening ear, offer reassurance, and assist when needed can make an immense difference in a mother's emotional well-being. Confiding in someone who understands and empathises with her struggles can alleviate the sense of isolation that often accompanies these disorders. Family and friends can offer a safe space for mothers to share their feelings without judgment, helping them process their emotions and navigate the challenges of motherhood. Additionally, practical help, such as babysitting, preparing meals, or running errands, can lighten the load and allow mothers the time and energy to focus on their recovery. The support of loved ones eases the emotional strain and reinforces the sense of community and belonging, reminding mothers that they are not alone on this journey [9].

Cultural and societal influences

Cultural Variations in Perceptions of Motherhood and Mental Health

The diverse tapestry of cultures across the globe contributes to a fascinating array of perspectives on motherhood and mental health. Cultural norms, traditions, and beliefs play an influential role in shaping how these topics are understood, discussed, and approached. Such cultural intricacies profoundly affect how different societies perceive and manage postpartum mood disorders [39].

In the realm of motherhood, cultural variations become evident in the expectations set for women as they embark on this transformative journey. Some cultures uphold a view of motherhood that idealises self-sacrifice and unconditional devotion to the family unit. In these societies, the role of a mother might be perceived as secondary to the well-being of her children and partner. Conversely, other cultures adopt a more holistic approach that recognises the importance of a mother’s well-being alongside her caregiving responsibilities. Here, the balance between self-care and caregiving is emphasised, acknowledging that a mother’s emotional and mental health directly impacts her ability to nurture her family effectively [40].

However, cultural norms can also give rise to challenges when acknowledging and addressing postpartum mood disorders. The stigmatisation of mental health issues might prevail in certain cultures, rendering discussions about emotional struggles taboo. This stigma can create a barrier that discourages mothers from openly expressing their symptoms or seeking professional help. The fear of being labelled as “weak” or “unfit” may lead to the underreporting of symptoms and a reluctance to access necessary support systems [41].

Understanding these cultural variations is pivotal for delivering effective and culturally sensitive care. Healthcare providers must be attuned to the nuances of each culture, recognising how perceptions of motherhood and mental health intersect. This awareness allows for developing strategies that break down barriers, encourage open conversations, and provide tailored support to mothers in a way that respects their cultural backgrounds. By embracing these diversities, we can bridge the gap between awareness and destigmatisation, ensuring that all mothers receive the comprehensive care they need while respecting their cultural contexts [42].

Barriers to Seeking Help in Different Societies

Cultural and societal factors can create significant barriers to seeking help for postpartum mood disorders, impeding the well-being of mothers and hindering timely interventions. These barriers are shaped by prevailing attitudes, norms, and expectations within specific societies, impacting how women perceive and address their mental health challenges [43].

Stigma around mental health issues: The mental health stigma is deeply entrenched in certain societies. Mental health issues may be misunderstood or associated with personal weakness or instability. Consequently, women experiencing postpartum mood disorders might feel ashamed or afraid of being judged by their communities. The fear of being labelled “mentally ill” can discourage women from acknowledging their struggles and seeking professional support [44].

Cultural notions of womanhood and caregiving: A woman’s role as a primary caregiver is often central in many cultures. Women are expected to prioritise the needs of their families and children, often at the expense of their well-being. Admitting emotional difficulties might be perceived as a deviation from this role, and women might worry that seeking help would be seen as a failure to fulfil their societal obligations. This clash between cultural expectations and emotional challenges can deter women from seeking desperately needed support [45].

These barriers highlight the complexity of addressing maternal mental health globally. Healthcare systems, governments, and communities must acknowledge and address these cultural and societal factors when designing interventions and support networks. By promoting awareness, education, and destigmatisation, we can gradually break down these barriers and create an environment where women feel empowered to seek help without fear of judgment or reproach.

Importance of Destigmatizing Postpartum Mood Disorders

The significance of destigmatising postpartum mood disorders cannot be overstated, as it holds profound implications for both individual well-being and the broader societal fabric. This process is crucial for a multitude of reasons that resonate deeply with the challenges faced by mothers:

Stigma prevents mothers from seeking help, delaying intervention and exacerbating emotional distress: The stigma surrounding mental health issues often casts a shadow of shame and embarrassment, leading mothers to internalise their struggles and refrain from seeking professional help. This delay in seeking intervention can prolong their emotional turmoil, potentially worsening symptoms and impeding their ability to care for themselves and their newborns effectively [46].

Promoting open conversations around mental health reduces isolation and fosters community support: Destigmatization paves the way for open discussions about postpartum mood disorders. When mothers feel empowered to share their experiences without fear of judgment, it breaks down the isolation that often accompanies these disorders. Open conversations foster a sense of belonging and validate the experiences of countless mothers who may be silently grappling with similar challenges [19].

Breaking down stigma encourages governments, healthcare systems, and communities to allocate resources for maternal mental health support: The weight of stigma extends beyond individual experiences. It obstructs progress on a systemic level, hindering governments, healthcare systems, and communities from prioritising maternal mental health support. By destigmatising these disorders, society can channel resources toward comprehensive and accessible care, ranging from awareness campaigns to specialised healthcare services catering to mothers’ unique needs [47].

Future directions and research

Advances in Understanding Hormonal Influences

As research continues, advances in understanding the intricate hormonal changes that occur during pregnancy and the postpartum period can shed light on the links between these fluctuations and mood disorders. A more profound comprehension of how hormonal shifts affect neurotransmitter regulation and brain function may pave the way for more targeted interventions and treatments.

Long-Term Effects on Maternal Mental Health

Exploring the long-term impact of untreated postpartum mood disorders on maternal mental health is an emerging area of research. Understanding how these disorders can influence a woman’s emotional well-being can highlight the necessity of early intervention and comprehensive support during the postpartum period.

Development of Targeted Interventions

*A*dvancements in personalised medicine offer the potential for tailoring interventions to individual needs. Genetic markers, hormonal profiles, and other factors can guide the development of more effective treatments for specific individuals, increasing the likelihood of positive outcomes.

## Conclusions

This comprehensive exploration underscores a vital call to action. Increased awareness and support are imperative to shatter the stigma enveloping postpartum mood disorders, encouraging mothers to seek assistance without hesitation. Collaborative efforts involving healthcare providers, communities, and governments are necessary to establish accessible and culturally sensitive support networks. Partners, family, and friends are pivotal in nurturing open dialogues and actively participating in the well-being of new mothers. Ultimately, a hopeful vision emerges - a future where maternal mental health is paramount. A future where no mother stands alone, where comprehensive resources and care empower them to navigate the complexities of motherhood with resilience. Through collective dedication, we can foster healthier mothers, strengthen familial bonds, and create communities that embody empathy and understanding. This review asserts that with concerted endeavours, we can transform this vision into reality, ensuring that every mother receives the appropriate mental health care she deserves.
